# Molecular Characterisation of Orf Virus in Goats From Eastern Türkiye

**DOI:** 10.1002/vms3.71044

**Published:** 2026-06-19

**Authors:** Remziye Özbek, Mehmet Ilgın, Kezban Can‐Şahna

**Affiliations:** ^1^ Faculty of Veterinary Medicine Department of Virology Sivas Cumhuriyet University Sivas Türkiye; ^2^ Elazığ Veterinary Control Institute Elazığ Türkiye; ^3^ Faculty of Veterinary Medicine Department of Virology Dokuz Eylül University İzmir Türkiye

**Keywords:** B2L, F1L, goat, molecular characterisation, Orf virus, Türkiye, VIR

## Abstract

**Background and aim:**

Orf virus (ORFV) is the etiologic agent of infectious ecthyma, a common worldwide disease that occasionally causes zoonotic infections. In this study, we aimed to detect and molecularly characterise circulating ORFV strains in goats from different provinces in eastern Türkiye.

**Materials and methods:**

Skin lesion samples collected during ORFV outbreaks between 2022 and 2024 were analysed. Following DNA extraction, positive samples identified by real‐time PCR were analysed using a multi‐locus phylogenetic approach targeting the structural *F1L*, the major envelope protein *B2L*, and the virulence‐related *VIR* gene regions. Sequencing was performed using the Sanger method, followed by phylogenetic analysis using the Maximum Likelihood method in MEGA X.

**Results:**

Phylogenetic analysis revealed high genetic similarity (95.63% to 99.48%) between the Turkish strains and global isolates from countries such as China, India, Iran, and Malaysia. Notably, while most isolates shared 100% similarity, the isolate from one specific province (Tunceli) exhibited significant nucleotide substitutions and a separate clustering pattern, particularly in the *VIR* gene.

**Conclusion:**

The results demonstrate that ORFV strains circulating in Türkiye display close phylogenetic clustering with Asian strains. The study highlights that multi‐locus analyses, especially utilising the highly variable *VIR* gene, are essential for identifying regional genetic heterogeneity and monitoring microbial evolution. These findings provide a pilot reference for future epidemiological surveillance and vaccine development strategies in the region.

## Graphical abstract

This study provides the molecular characterisation of Orf virus (ORFV) strains circulating in Türkiye. Phylogenetic analysis of *F1L, B2L*, and *VIR* genes reveals high genetic similarity with Asian and global isolates, highlighting the importance of multi‐gene analysis in monitoring viral outbreaks and understanding disease origins.

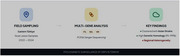

## 1 Introduction

Orf virus (ORFV) is the etiological agent of contagious ecthyma, also known as contagious pustular dermatitis, sore mouth, scabby mouth, or orf. This disease is widespread globally and causes significant economic losses (Efridi et al. 2023; Spyrou and Valiakos 2015). This virus is globally endemic in small ruminants, particularly sheep and goats, across all regions where these animals are raised, and can occasionally infect wild ruminant species (Azwai et al. 1995; Coradduzza et al. 2024). It is characterised by proliferative lesions affecting the skin of the lips, the perinasal region, and the oral mucosa (Mondal et al. 2006). Lesions typically develop through stages of erythema, papules, pustules, and scab formation, and usually heal within 1–2 months. Although the disease is generally considered mild, mortality rates of up to 93% have been reported in goat kids. Lesions on the lips and udders prevent infected animals from suckling and grazing, leading to rapid weight loss and high mortality (Delhon et al. 2004; Ma et al. 2022). Due to the zoonotic nature of the disease, humans are at high risk of infection, which typically occurs through direct contact with infected animals or indirectly via fomites contaminated with the ORFV (Caravaglio and Khachemoune 2017; Mahmud et al. 2014).

Orf virus is the prototype species of the genus *Parapoxvirus*, which belongs to the subfamily *Chordopoxvirinae* within the family *Poxviridae* (Wittek et al. 1979). ORFV possesses a linear double‐stranded DNA genome of approximately 140 kb and forms virions measuring nearly 260 × 160 nm. In contrast to other members of the *Poxviridae* family, the ORFV genome exhibits a relatively high G+C content (63%–64%) and contains 132 predicted open reading frames. Genomic organisation is characterised by a conserved central region, whereas more variable genes are predominantly located at the terminal regions. Genes involved in viral DNA replication and cytoplasmic particle assembly are located in the central genomic region, whereas the terminal regions predominantly encode virulence‐related genes that are not essential for viral replication (Mahmud et al. 2014; Mercer et al. 2006; Nandi et al. 2011). Among these, the most frequently used target genes for ORFV genetic variation and molecular epidemiological studies are *F1L (ORFV059)*, major viral envelope protein *B2L (ORFV011)*, *DNA polymerase*, *GIF/IL‐2*, and virus interferon resistance *VIR (ORFV020)* genes (Hussain et al. 2022; Pang and Long 2023).

The *F1L* gene encodes an envelope‐associated immunogenic protein that contributes to viral attachment and entry by interacting with heparan sulphate receptors. The *B2L* gene encodes a major envelope protein with lipase activity and strong immunogenic properties. The *VIR* gene is associated with viral interferon resistance and encodes a double‐stranded RNA (dsRNA)–binding protein that modulates host interferon‐mediated antiviral responses during infection (Li et al. 2023).

Despite the presence of characteristic lesions on the lips and oral mucosa in ORFV infections, laboratory‐based methods are essential to confirm the diagnosis and to support epidemiological investigations (Yang et al. 2015). Molecular diagnostic approaches for ORFV include PCR‐based methods such as conventional PCR and real‐time PCR, as well as isothermal amplification techniques including loop‐mediated isothermal amplification, recombinase polymerase amplification, and recombinase‐aided amplification assays (Pang and Long 2023).

In this study, we aimed to detect and molecularly characterise circulating ORFV strains in goats from different provinces in eastern Türkiye. This characterisation was based on a multi‐locus phylogenetic approach, integrating the structural and immunogenic properties of the *F1L* and *B2L* regions with the high genetic variability of the virulence‐related *VIR* gene to achieve a high‐resolution molecular profile of the field isolates.

## 2 Materials and Methods

### 2.1 Sampling

The study material consisted of skin lesion samples collected from animals with suspected ORF that were submitted to the Republic of Türkiye Ministry of Agriculture and Forestry, Elazığ Veterinary Control Institute, from the provinces of Elazığ, Diyarbakır, and Tunceli between 2022 and 2024.

Samples were collected from hair goat (*Capra hircu*s) herds. The affected animals were primarily kids with an average age of 3 to 4 months. Clinical severity was most pronounced in this age group, characterised by facial oedema, anorexia, and a mortality of 13 kids in one herd. In adult goats, lesions were localised primarily on the udders, suggesting a transmission link between nursing kids and dams. These epidemiological findings, along with the lack of response to prior oxytetracycline and enrofloxacin treatments, were recorded during the field study.

### 2.2 DNA Extraction

Skin papule samples were collected in 2 mL sterile Eppendorf tubes. The samples were suspended in 10%–20% PBS (Phosphate Buffer Saline) and homogenised using a homogeniser. After vortexing, the samples were centrifuged at 4°C and 3000 rpm for 5 min. The supernatants were then transferred to sterile stock tubes for extraction. The supernatants were stored at –20°C until extraction.

Viral DNA extraction was carried out using the QIAGEN QIAcube instrument and the IndiSpin Pathogen Nucleic Acid Isolation Kit (Cat. No. SP54106), in accordance with the manufacturer's instructions.

### 2.3 Real‐Time PCR

Real‐time PCR was performed targeting the ORFV *DNA polymerase (pol) gene*, following the protocol described by Bora et al. (2011). A GoTaq Probe qPCR Master Mix (Cat. No.: A6102) and a QIAGEN/Rotor‐Gene Q device were used for DNA amplification. For the Real‐time PCR step, a total reaction volume of 20 µL was prepared containing 1 µL of template DNA, 1 µL of each primer (10 µM), 1 µL of probe (5 µM), 10 µL of 2X Master Mix, and 6 µL of nuclease‐free water. To ensure diagnostic validity, a positive control (confirmed ORFV DNA), a no‐template negative control, and an extraction control were included in each run. Samples were considered positive if they exhibited a characteristic amplification curve with a cycle threshold (Ct) value < 35.

### 2.4 PCR

Seven samples that tested positive by real‐time PCR were subjected to conventional PCR analysis using primers designed by Yang et al. (2014) targeting the *B2L, F1L*, and *VIR* gene regions, following the same protocol. For amplification, the commercial kit Thermo Scientific‐ PCR Master Mix 2X (Cat. No.: K0171) was used. For each sample, a reaction mixture was prepared with a total volume of 25 µL containing 12.5 µL of 2X PCR Master Mix, 1 µL of forward and reverse primers each (10 µM), 1 µL of template DNA, and 9.5 µL of deionised water.

To visualise PCR amplicons, a 1% agarose gel containing GelRed (Biotium, USA) was prepared. The amplicons were mixed with loading dye (6× Loading Dye, Thermo Scientific, Lithuania) and loaded into the gel wells. A 100 base pairs (bp) DNA ladder (Thermo Scientific, Lithuania) was used to estimate product sizes, and electrophoresis was performed at 16 V/cm for approximately 30 min, after which the bands were visualised under UV light.

### 2.5 Sequence Analysis

Sequencing analysis of the PCR products targeting the *B2L, F1L*, and *VIR* gene regions was performed on four samples, selected from seven positive samples to represent different provinces, by a commercial service provider (Innopenta Biotechnology Inc., Ankara, Türkiye) using the Sanger sequencing method.

Sequence chromatograms were examined using FinchTV version 1.4.0 (Geospiza Inc., Seattle, WA, USA). The raw sequences were initially evaluated based on chromatogram quality, and sequence trimming was performed to remove primer sequences and low‐quality terminal regions. The resulting high‐quality sequences were then identified and compared with previously published data using the BLASTn tool at the National Center for Biotechnology Information (NCBI). Subsequently, the processed sequences were imported into MEGA X software for further analyses (Kumar et al. 2018). Sequence alignment was carried out using reference sequences, and nucleotide variations were identified based on comparisons with these references.

### 2.6 Phylogenetic Analysis

**FIGURE 1:**
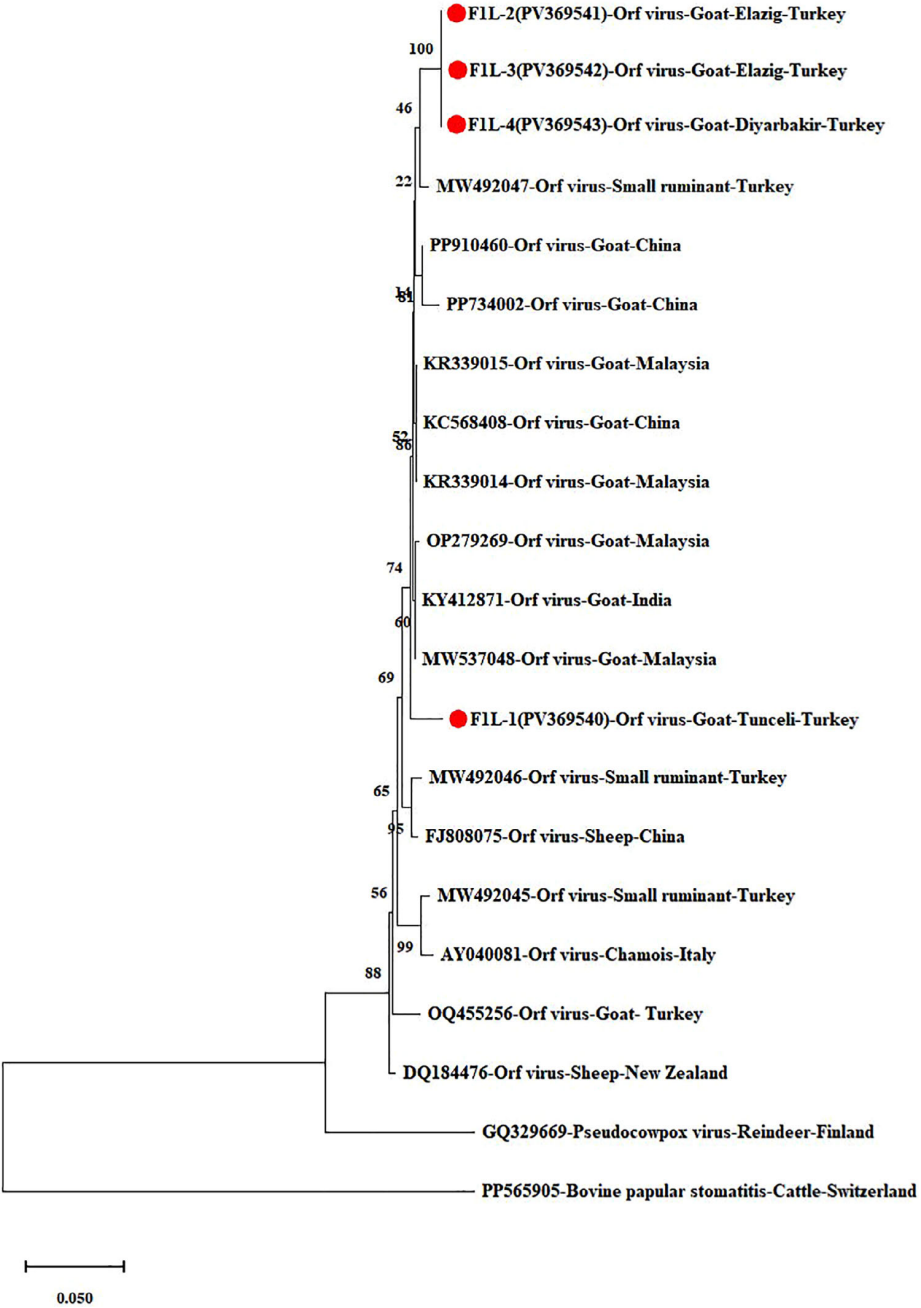
Phylogenetic tree based on the *F1L* gene (the circles with red centres represent the Turkish isolates obtained from this study).

**FIGURE 2:**
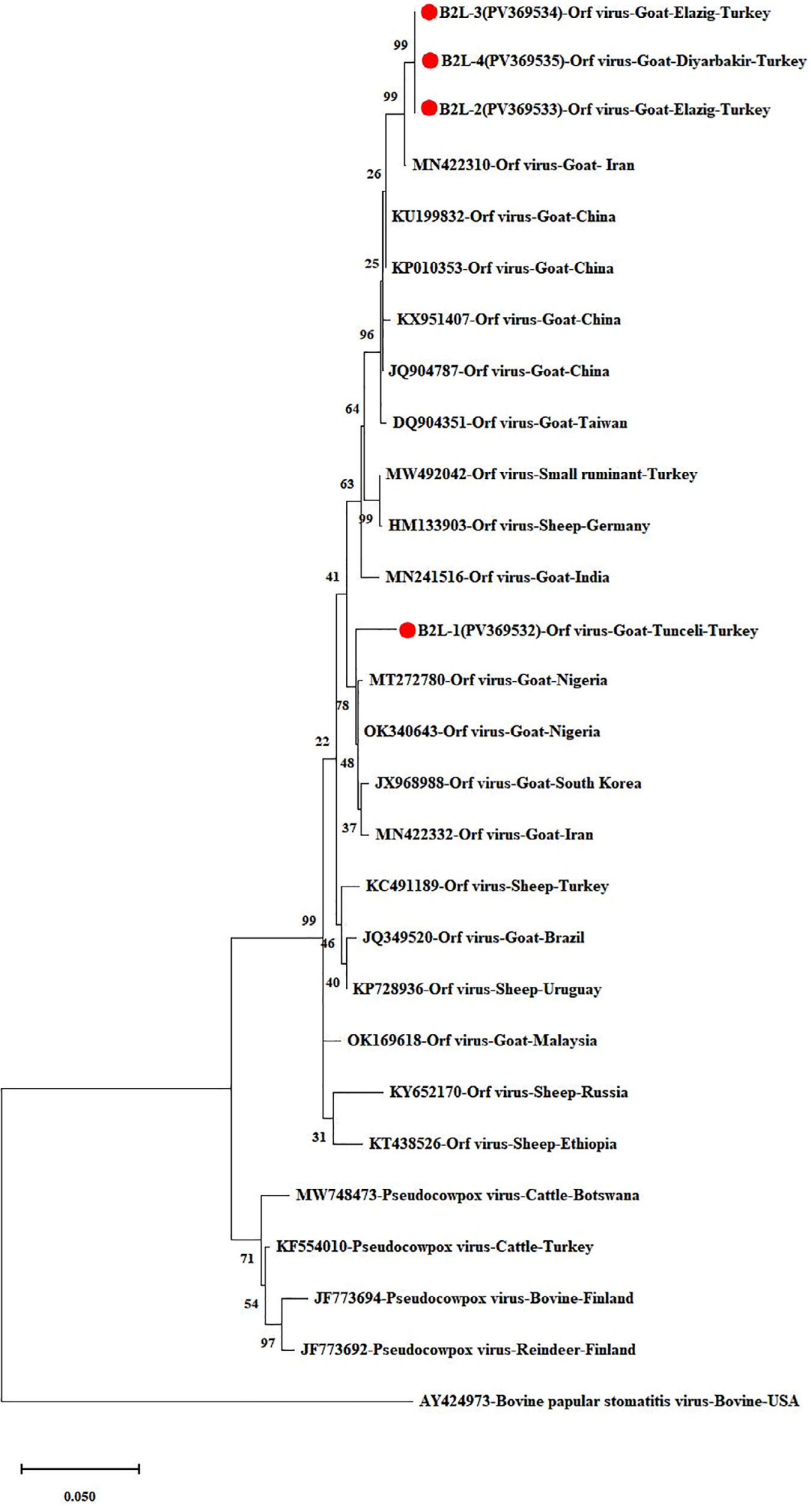
Phylogenetic tree based on the *B2L* gene (the circles with red centres represent the Turkish isolates obtained from this study).

**FIGURE 3:**
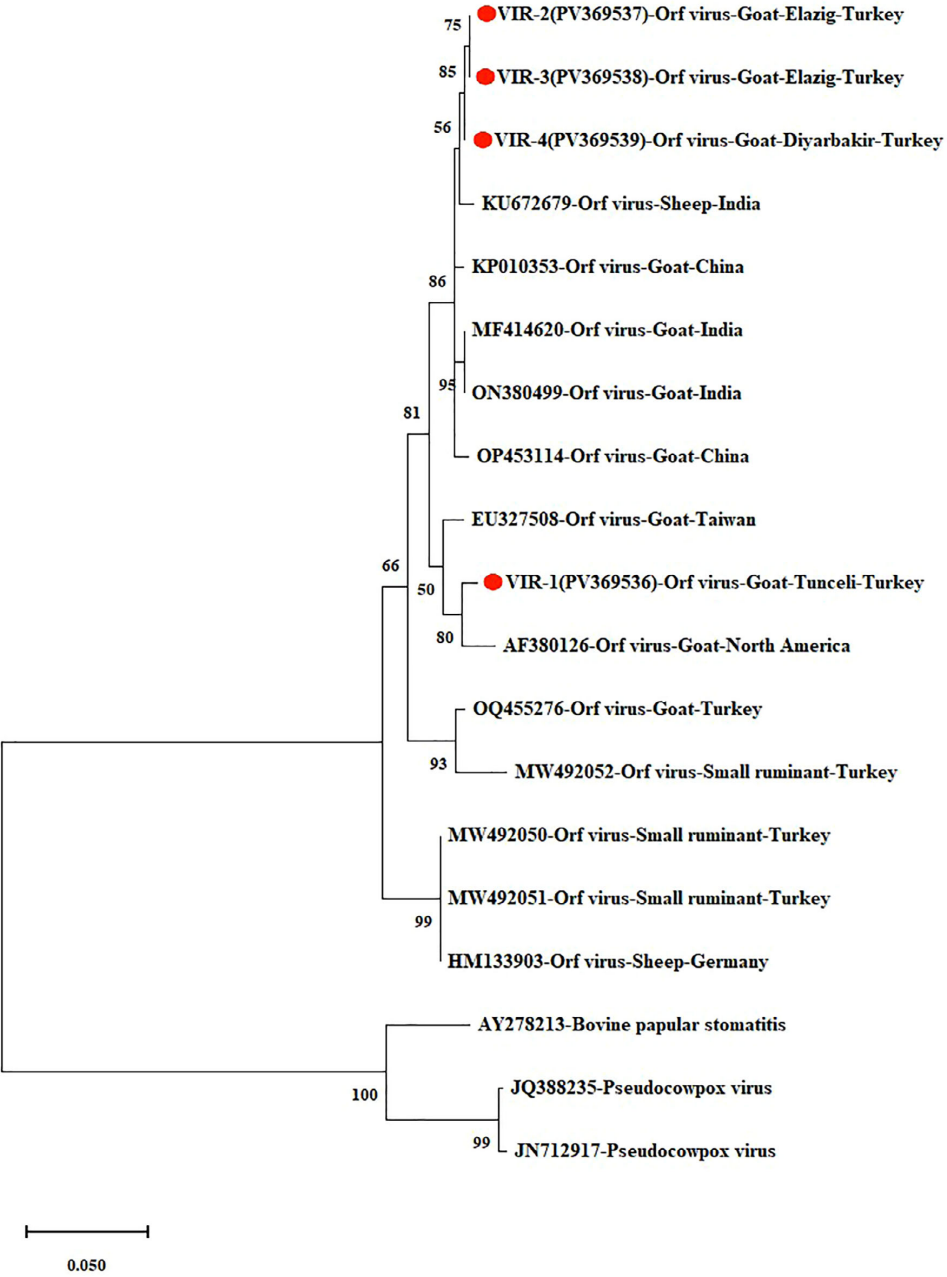
Phylogenetic tree based on the *VIR* gene (the circles with red centres represent the Turkish isolates obtained from this study).

For phylogenetic analysis, reference sequences retrieved from NCBI were included as outgroups (Figures 1, 2, 3). Multiple sequence alignment was conducted using the ClustalW algorithm implemented in MEGA X. The most suitable phylogenetic tree model for the sequences was determined as the Tamura 3‐parameter model with Gamma distribution (T92+G), and the evolutionary tree was constructed using the Maximum Likelihood statistical method with bootstrap analysis (1000 replicates) (Kumar et al. 2018). Bootstrap values ≥70% were considered to indicate statistically significant node support. To ensure proper tree rooting, closely related members of the genus *Parapoxvirus*, includ**i**ng *pseudocowpox* *virus* and *bovine papular stomatitis virus*, were selected as outgroup sequences.

## 3 Results

In this study, partial sequencing and phylogenetic analysis of the ORFV *F1L*, *B2L*, and *VIR* gene regions were conducted to determine the genetic and evolutionary relationships among Turkish ORFV isolates and globally circulating ORFV strains, as well as other closely related members of the *Parapoxvirus* genus. The Turkish isolates analysed in this study are summarised in Table 1.

**TABLE 1:** Turkish ORFV isolates analysed in this study.

Isolate	Gene	GenBank Accession No.	Region/source
F1L‐1	F1L	PV369540.1	Tunceli/Türkiye
F1L‐2	F1L	PV369541.1	Elazığ/Türkiye
F1L‐3	F1L	PV369542.1	Elazığ/Türkiye
F1L‐4	F1L	PV369543.1	Diyarbakır/Türkiye
B2L‐1	B2L	PV369532.1	Tunceli/Türkiye
B2L‐2	B2L	PV369533.1	Elazığ/Türkiye
B2L‐3	B2L	PV369534.1	Elazığ/Türkiye
B2L‐4	B2L	PV369535.1	Diyarbakır/Türkiye
VIR‐1	VIR	PV369536.1	Tunceli/Türkiye
VIR‐2	VIR	PV369537.1	Elazığ/Türkiye
VIR‐3	VIR	PV369538.1	Elazığ/Türkiye
VIR‐4	VIR	PV369539.1	Diyarbakır/Türkiye

PCR analysis yielded amplicons of 1023 bp, 1137 bp, and 552 bp corresponding to the *F1L, B2L*, and *VIR* genes, respectively.

Partial sequence analysis of the 808 bp ORFV *F1L* gene demonstrated nucleotide differences between Turkish isolates (F1L‐1, F1L‐2, F1L‐3, and F1L‐4) and the reference strain (MW537048). Isolates F1L‐2, F1L‐3, and F1L‐4 displayed the same variation pattern, whereas F1L‐1 showed additional nucleotide substitutions (Table 2).

**TABLE 2:** Nucleotide variation position of *F1L* gene (808 bp).

Nucleotide position	16	187	188	249	288	308	316	385	424	427	439	457	469	472	490	514	533	534	550	574	694	756	760	762
MW537048*	C	T	G	G	G	G	C	C	C	C	C	C	G	T	C	A	G	C	C	C	C	T	C	T
F1L‐1		C	A		A			T		T		G	A		T	G		A		T	T	G	G	G
F1L‐2	T			A	A	A	T		A		T			G			A		T				G	
F1L‐3	T			A	A	A	T		A		T			G			A		T				G	
F1L‐4	T			A	A	A	T		A		T			G			A		T				G	

A: adenine, T: thymine, C: cytosine, G: guanine.

*Reference sequence.

Molecular characterisation and phylogenetic analysis of the ORFV *F1L* gene demonstrated 100% nucleotide similarity among some Turkish isolates (F1L‐2, F1L‐3, F1L‐4) obtained from this study, and high similarity (98.64%) to strains/isolates from China (KC568408), Malaysia (KR339014, KR339015, MW537048, OP279269), and India (KY412871). The F1L‐1 isolate obtained from the study was placed in a separate cluster, exhibiting 98.14% similarity with other Turkish strains and 97.9% similarity with the Malaysian isolate (OP279269). Sequence similarity between the isolates included in this study and globally circulating goat ORFV strains retrieved from GenBank ranged from 96.29% to 98.64%. In contrast, similarity with other parapoxviruses, including pseudocowpox virus and bovine papular stomatitis virus, ranged from 75.2% to 90.9% (Figure 1).

Comparative analysis of the 963 bp partial sequences of the ORFV B2L gene showed nucleotide‐level differences between Turkish ORFV isolates (B2L‐1, B2L‐2, B2L‐3, and B2L‐4) and the reference strain (KP010353). A high degree of similarity was observed among the analysed isolates; B2L‐2, B2L‐3, and B2L‐4 exhibited broadly similar variation profiles, whereas B2L‐1 showed limited divergence from this group (Table 3).

**TABLE 3:** Nucleotide variation position of *B2L* gene (963 bp).

Nucleotide position	55	67	70	118	133	138	172	181	220	277	278	279	283	298	314	316	325	329	358	376	469	500	520	562	586	787	840	849	861	873	901	907	921	949
KP010353*	C	C	C	G	G	T	G	C	G	G	C	G	A	T	A	A	C	C	T	T	A	G	G	G	C	G	T	T	T	T	C	C	T	C
B2L‐1	T	T		T		C		A		C	A	A	G	G	C	C		A	G	C	G	A		A		A	C	G	G	G	A	T	G	
B2L‐2			T	A	A		A		A								T				G		A		T					C				A
B2L‐3			T	A	A		A		A								T				G		A		T					C				A
B2L‐4			T	A	A		A		A								T				G		A		T					C				A

A: adenine, T: thymine, C: cytosine, G: guanine.

*Reference sequence.

The B2L‐2, B2L‐3, and B2L‐4 isolates exhibited 100% nucleotide identity with each other and shared 99.48% similarity with the Iranian isolate (MN422310). The B2L‐1 isolate identified in this study showed 96.57% similarity with the other Turkish isolates included in the analysis. In addition, it exhibited high sequence similarity (97.92%–98.34%) with ORFV strains/isolates from Nigeria (MT272780, OK340643), Korea (JX968988), and Iran (MN422332), clustering within the same phylogenetic group. Overall, similarity rates between the isolates analysed in this study and other goat ORFV strains/isolates selected from GenBank ranged from 96.56% to 99.48%. In comparison, similarity with other parapoxvirus isolates ranged from 83.39% to 97.82% (Figure 2).

Comparative analysis of 503 bp partial sequences of the VIR gene revealed significant nucleotide differences between ORFV isolates of Turkish origin (VIR‐1, VIR‐2, VIR‐3, and VIR‐4) and the reference strain (ON380499). Consistent nucleotide substitutions were identified in all isolates compared with the reference strain. The VIR‐2 and VIR‐3 isolates shared identical substitution patterns, whereas the VIR‐1 isolate exhibited partial divergence from this pattern. The VIR‐4 isolate had fewer nucleotide substitutions than the other isolates (Table 4).

**TABLE 4:** Nucleotide variation position of VIR gene (503 bp).

Nucleotide position	29	53	65	72	92	94	97	102	137	141	203	258	308	320	327	417	466
ON380499*	G	C	T	G	C	C	G	A	C	G	C	T	C	G	C	G	A
VIR‐1		T	G	C	A	T	A	G	T		T	G	T	A		T	G
VIR‐2	T				A					A					T		G
VIR‐3	T				A					A					T		G
VIR‐4	T				A										T		G

A: adenine, T: thymine, C: cytosine, G: guanine.

*Reference sequence.

The VIR‐2, VIR‐3, and VIR‐4 isolates obtained in this study exhibited 99.8%–100% nucleotide similarity within the VIR gene region. Furthermore, these isolates showed high nucleotide similarity (98.81%–99.01%) with strains/isolates from China (KP010353, OP453114) and India (MF414620, ON380499, KU672679). The VIR‐1 isolate obtained in this study showed 97.02–97.22% nucleotide similarity with other Turkish strains, with the highest similarity to strains/isolates from the United States (AF380126) (98.01%) and Taiwan (EU327508) (97.81%), and clustered within the same phylogenetic group. Overall, the isolates from this study displayed nucleotide similarity rates of 95.63%–99.01% with other goat ORFV strains and isolates in GenBank and 75.64%–99.01% with other parapoxvirus isolates (Figure 3).

## 4 Discussion

In this study, phylogenetic trees were constructed to assess the genetic relationships between goat ORFVs from different regions of Türkiye, selected global strains, and related parapoxviruses. To provide a more granular perspective on regional diversity, this study utilised a multi‐locus approach by simultaneously analysing the F1L, B2L, and VIR genes. While our findings align with the general prevalence of Asian‐like strains in the country, the use of multi‐locus analyses allowed us to identify subtle but significant intra‐regional variations. In contrast to the field isolates from Türkiye found in the other two provinces, which shared 100% nucleotide similarity, the Tunceli isolate (F1L‐1, B2L‐1, VIR‐1) exhibited remarkable nucleotide changes. The nucleotide similarity of the Tunceli isolate ranged from 96.57% to 98.64% relative to the Elazığ and Diyarbakır isolates, serving as an indicator of localised evolutionary dynamics and genetic heterogeneity. This suggests that even within geographically close areas, ORFV can exhibit unique clustering patterns. Therefore, this study demonstrates that multi‐locus analyses are a useful approach for uncovering the degree of genetic diversity that might be overlooked in single‐gene surveillance studies.

ORFV is currently endemic in Türkiye, and outbreaks in goats are increasing (Kaplan et al. 2023). Although various phylogenetic analyses of ORFV cases in Türkiye have been conducted in recent years (Durmuş et al. 2025; Karapınar and Gürses 2024; Koç 2022; Yıldırım et al. 2025), high‐resolution genomic data characterising the virus remain limited. Our study aims to complement this growing body of literature by providing updated molecular insights into currently circulating strains.

For genetic and evolutionary analyses as well as molecular epidemiological studies of ORFV, the *B2L* and *F1L* genes are most commonly targeted (Billinis et al. 2012; Yu et al. 2020). These genes are highly conserved in ORFV and therefore may not fully represent the actual genetic diversity among different ORFV strains. Consequently, relying solely on *B2L* and *F1L* genes may be insufficient to comprehensively characterise the genetic variability of ORFV. To obtain a more accurate assessment of the genetic characteristics and evolutionary dynamics of ORFV strains, additional genomic regions should be analysed alongside *B2L* and *F1L*. In previous studies, other genes such as *ORF020 (VIR)*, *GIF, vIL‐10, ORF109, ORF110, ORF117, ORF119, ORF125*, and *ORF127* have also been used (Li et al. 2023; Zhang et al. 2024).

The *F1L* gene of ORFV is located in the 59th open reading frame of the viral genome and contributes to viral entry into host cells through binding to heparan sulphate receptors (Gallina et al. 2004; Scagliarini et al. 2004). The protein encoded by this gene is a major antigen that can induce both cellular and humoral immune responses. The *F1L* gene is among the most important genes for assessing strain variation (Lin et al. 2024; Yogisharadhya et al. 2018). Four ORFV isolates from this study were aligned with 17 strains currently available in GenBank, and a phylogenetic tree was constructed. Sequence data and phylogenetic analyses based on the *F1L* gene showed that the isolates from this study had high homology (97.9%–98.64%) with strains/isolates from China, India, and Malaysia.

The B2L gene of ORFV encodes a highly immunogenic envelope protein that induces a strong antibody response (Wang et al. 2023). This gene is widely used as a PCR target for ORFV detection and is also employed in phylogenetic analyses due to its specificity and conservation within ORFV (Zhang et al. 2014). Phylogenetic analysis was conducted based on sequence alignments of four ORFV isolates from the present study and 24 ORFV strains downloaded from GenBank. Sequence data and phylogenetic analyses based on the B2L gene revealed that the isolates in this study are closely related (97.92%–99.48%) to strains/isolates from Iran, Nigeria, and Korea. Recent studies on the B2L gene in Türkiye (Karapınar and Gürses 2024; Yıldırım et al. 2025) have reported that Turkish strains show high similarity rates with Asian and African strains. While our study correlates with these studies in that the majority of strains are similar to those from Asia, heterogeneity was also observed.

The ORFV interferon resistance protein (VIR) is a major virulence factor that encodes a dsRNA‐binding protein that inhibits the host antiviral response (Yan et al. 2020). Yan et al. (2020) demonstrated that the ORFV VIR protein interacts with the host cell–derived p53 protein, which plays a key role in immune responses, and promotes its degradation, thereby inhibiting p53‐mediated positive regulation. Molecular studies of the ORFV genome have revealed that the *VIR* gene undergoes higher evolutionary change (mutation) than other genomic regions; this is an essential indicator for understanding the virus's genetic diversity and adaptation processes (Li et al. 2023). The high mutation rate observed in the *VIR* gene can be interpreted as a reflection of the virus's evasion from host interferon responses and its adaptation strategies; this makes the *VIR* gene a more sensitive marker than *B2L* and *F1L* for monitoring microbial evolution. Therefore, potential mutations occurring in this gene may be necessary for the development of disease control and prevention strategies, particularly vaccine studies. For this reason, up‐to‐date molecular and phylogenetic analyses of the *VIR* gene of currently circulating strains in the country are required. In a study conducted in different regions of Türkiye focusing on the *VIR* gene (Durmuş et al. 2025), Turkish ORFV strains were reported to exhibit close genetic similarity to strains originating from China, Argentina, and India. Similarly, phylogenetic analyses of the *VIR* gene in this study revealed that current Turkish isolates are closely related to strains/isolates from China, India, the United States, and Taiwan, exhibiting high nucleotide similarity (98.81%–99.01%).

From an epidemiological and evolutionary perspective, the high degree of similarity observed between the current isolates and various Asian strains potentially offers broader insights into the viral evolution within the region. These molecular correlations could be attributed to recent transmission events, while concurrently serving as a possible indicator of shared ancestral lineages or historical livestock trade routes that may have linked these geographical areas over extended periods. Furthermore, leveraging the higher genetic variability of the VIR gene, as opposed to the more conserved B2L and F1L regions, allows for a finer phylogenetic resolution in distinguishing these isolates. Utilising this variability may provide a more nuanced understanding of the micro‐evolutionary shifts, the viral dynamics, and the intricate genetic relatedness that likely exists among the ORFV strains currently circulating in Türkiye.

The translational relevance of such molecular characterisation extends beyond evolutionary analysis, playing a critical role in the management of the disease. Detailed genomic data on circulating strains could inform the strategic development of vaccines specifically tailored to the genetic landscape of the region. Furthermore, integrating these findings into national surveillance programs may enhance the efficiency of disease tracking. Given the zoonotic potential of ORFV, constant monitoring of genetic variations remains essential for assessing public health risks and strengthening cross‐species transmission surveillance.

In conclusion, this study provides a preliminary identification and molecular characterisation of ORFVs currently circulating in Eastern Türkiye. Although the limited number of samples may constrain the generalisability of the findings to larger populations, the genetic variations and phylogenetic clustering observed among the sequenced isolates indicate a degree of regional heterogeneity. These divergent points suggest that ORFV populations in the region might not follow a uniform genetic structure. Therefore, broader geographical sampling with larger sample sizes is required to more comprehensively capture the national genetic diversity and to determine if this variation is consistent across different provinces. Additionally, future directions should incorporate whole‐genome sequencing (WGS) to provide a more detailed resolution of the viral evolution. Nevertheless, these molecular and phylogenetic correlations may serve as a useful tool and a pilot reference for future epidemiological surveillance studies in Türkiye.

## Author Contributions

All authors have read and approved the manuscript. RO conceptualised the study and designed the experiments. Experiments were conducted by RÖ and MI. Data analysis, including preparation of tables and figures, was performed by KCS, who also contributed to visualisation and supervision. All authors contributed to the interpretation of the results, critically revised the manuscript, and approved the final version for publication.

## Funding

The authors have nothing to report.

## Ethics Statement

This study was conducted in accordance with the official authorisation (No. E‐71037622‐604.01.01‐18228292) obtained from the General Directorate of Food and Control of the Republic of Türkiye Ministry of Agriculture and Forestry and the ethical approval (Decision No. 2025/08‐01) issued by the Local Animal Ethics Committee of the Elazığ Veterinary Control Institute.

## Conflicts of Interest

The authors declare no conflicts of interest.

## Data Availability

All authors confirm that the data supporting the findings of this study are included in the main manuscript file.
